# Supplementary figures and images for: Associations between symptoms of sleep-disordered breathing and maternal sleep patterns with late stillbirth: Findings from an individual participant data meta-analysis
Source: PLoS One. 2020 Mar 26;15(3):e0230861. doi: 10.1371/journal.pone.0230861 (PMC7098581; doi:10.1371/journal.pone.0230861)

**S1 Fig: Chart of available data from contributing studies**


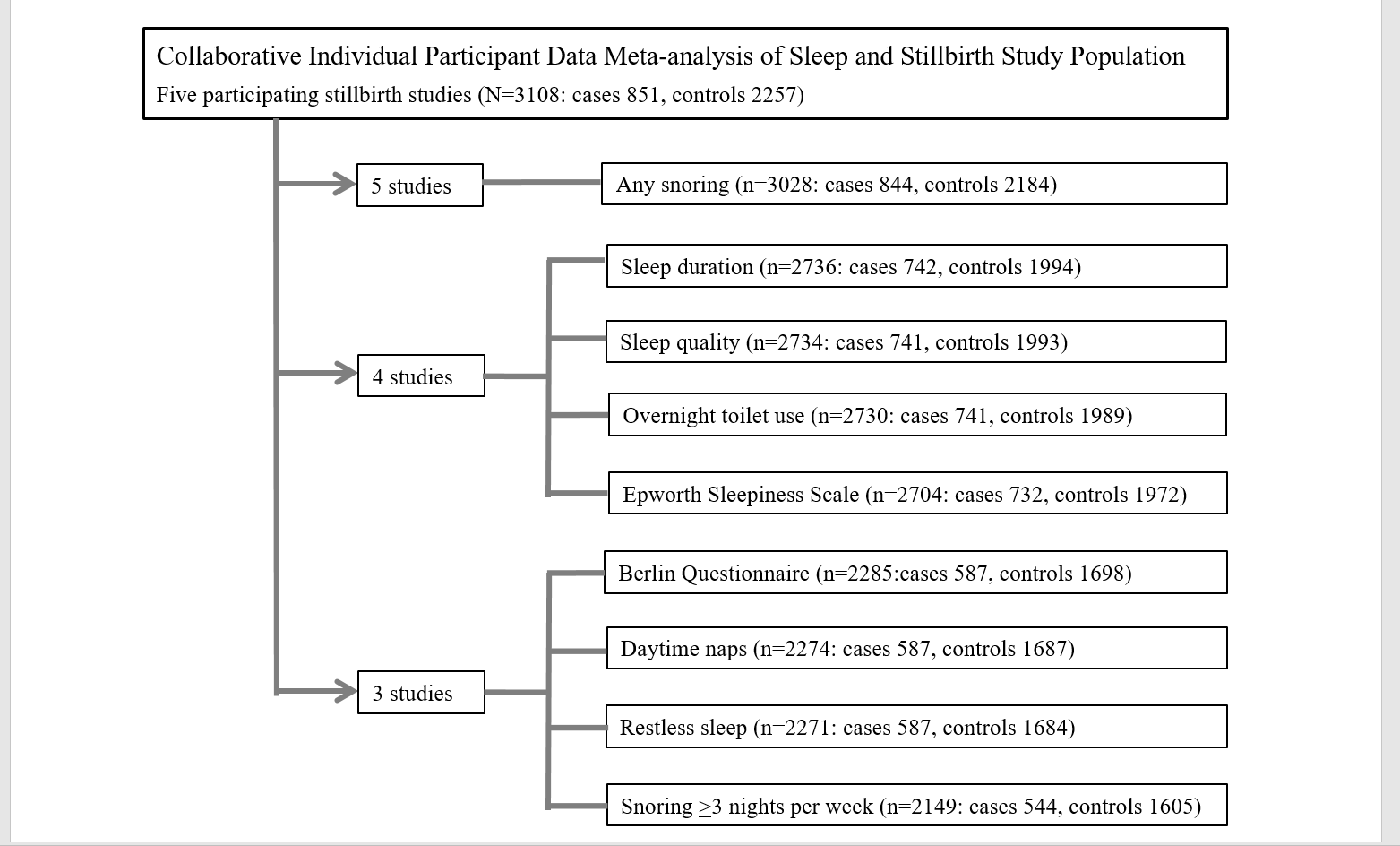

Supplement: S1 Fig — (DOCX) [file pone.0230861.s001.docx]
