# Supplementary material for: Associations between symptoms of sleep-disordered breathing and maternal sleep patterns with late stillbirth: Findings from an individual participant data meta-analysis
Source: PLoS One. 2020 Mar 26;15(3):e0230861. doi: 10.1371/journal.pone.0230861 (PMC7098581; doi:10.1371/journal.pone.0230861)
Supplement: S1 Table — (DOCX) [file pone.0230861.s002.docx]

**S1 Table: Participant level characteristics and non-sleep late stillbirth risk factors in participating case-control studies and pooled IPD meta-analysis.**

|  | **TASS**  **Stacey et al (2011)⁶** | | **SSS**  **Gordon et al (2015)⁹** | | **MCSS**  **McCowan et al (2017)⁴** | | **MiNESS**  **Heazell et al (2017)⁸** | | | **STARS**  **O’Brien et al (2018)⁷** | | | **Collaborative Individual Participant Data of Going-to-sleep and Stillbirth (CRIBSS) analysis** | | | |
| --- | --- | --- | --- | --- | --- | --- | --- | --- | --- | --- | --- | --- | --- | --- | --- | --- |
| **Characteristic** | Case | Control | Case | Control | Case | Control | | Case | Control | | Case | Control | Case | Control | Univariable  odds ratio  (95% CI) | Adjusted  odds ratio  (95% CI) |
| **Total participants** | 155 (33.8) | 304 (66.2) | 103 (34.9) | 192 (65.1) | 163 (22.5) | 560 (77.5) | | 288 (28.2) | 733 (71.8) | | 142 (23.3) | 468 (76.7) | 851 (27.4) | 2257 (72.6) |  |  |
| **Age (years)** | | | | | | | | | | | | | | | | |
| <20 | 10 (6.5) | 24 (7.9) | 3 (2.9) | 1 (0.5) | 9 (5.5) | 17 (3.0) | | 7 (2.4) | 15 (2.1) | | 9 (6.3) | 21 (4.5) | 38 (4.5) | 78 (3.5) | 1.50 (0.98-2.28) | 1.29 (0.61-2.74) |
| 20–24 | 29 (18.7) | 44 (14.5) | 4 (3.9) | 14 (7.3) | 23 (14.1) | 79 (14.1) | | 47 (16.3) | 81 (11.1) | | 12 (8.5) | 56 (12.0) | 115 (13.5) | 274 (12.1) | 1.33 (1.02-1.73) | 0.99 (0.63-1.54) |
| 25–29 | 40 (25.8) | 75 (24.7) | 25 (24.3) | 43 (22.4) | 40 (24.5) | 157 (28.0) | | 82 (28.5) | 219 (29.9) | | 41 (28.9) | 137 (29.3) | 228 (26.8) | 631 (28.0) | 1.12 (0.91-1.38) | 0.99 (0.71-1.37) |
| 30–34 | 44 (28.4) | 92 (30.3) | 39 (37.9) | 66 (34.4) | 48 (29.5) | 191 (34.1) | | 85 (29.5) | 268 (36.6) | | 49 (34.5) | 177 (37.8) | 265 (31.1) | 794 (35.2) | 1 | 1 |
| 35–39 | 29 (18.7) | 57 (18.8) | 25 (24.3) | 51 (26.6) | 29 (17.8) | 96 (17.1) | | 51 (17.7) | 125 (17.1) | | 26 (18.3) | 67 (14.3) | 160 (18.8) | 396 (17.6) | 1.19 (0.94-1.51) | 1.67 (1.16-2.40) |
| >40 | 3 (1.9) | 12 (4.0) | 7 (6.8) | 17 (8.9) | 14 (8.6) | 20 (3.6) | | 16 (5.6) | 25 (3.4) | | 5 (3.5) | 10 (2.1) | 45 (5.3) | 84 (3.7) | 1.60 (1.08-2.38) | 2.61 (1.45-4.70) |
| **Earliest pregnancy BMI (kg/m^2^ )** | 27.5 (23.2-33.3) | 25.0 (22.2–  30.8) | 23.1 (21.2–28.3) | 22.8 (20.8–  26) | 26.6 (23.1–33.5) | 24.8 (22.0–29.6) | | 26.1 (22.5–30.3) | 24.9 (22.1–28.8) | | 26.8 (22.8–32.5) | 25.2 (22.5–31.1) | 26.0 (22.5–31.4) | 24.8 (22.0–  29.3) | 1.04 (1.03-1.05) | 1.03 (1.01-1.05) |
| **Ethnicity** | | | | | | | | | | | | | | | | |
| White | 55 (35.5) | 134 (44.1) | 57 (55.3) | 136 (70.8) | 65 (39.9) | 260 (46.4) | | 233 (80.9) | 594 (81.0) | | 112 (78.9) | 421 (90.0) | 522 (61.3) | 1545 (68.5) | 1 | 1 |
| Black | 4 (2.6) | 1 (0.3) | 1 (1.0) | 3 (1.6) | 2 (1.3) | 3 (0.5) | | 12 (4.2) | 29 (4.0) | | 3 (2.1) | 6 (1.3) | 22 (2.6) | 42 (1.9) | 1.63 (0.94-2.84) | 1.60 (0.71-3.70) |
| South Asian* | 15 (9.7) | 27 (8.9) | 9 (8.7) | 10 (5.2) | 17 (10.4) | 85 (15.2) | | 38 (13.2) | 94 (12.8) | | 11 (7.8) | 3 (0.6) | 90 (10.6) | 219 (9.7) | 1.40 (1.04-1.88) | 2.13 (1.37-3.32) |
| South East/East Asian | 12 (7.7) | 25 (8.2) | 15 (14.6) | 18 (9.4) | 12 (7.4) | 64 (11.4) | | 1 (0.4) | 4 (0.6) | | - | - | 40 (4.7) | 111 (4.9) | 1.19 (0.8-1.79) | 0.99 (0.46-2.11) |
| Maori | 19 (12.3) | 46 (15.1) | 1 (1.0) | 3 (1.6) | 26 (16.0) | 58 (10.4) | | - | - | | - | - | 46 (5.4) | 107 (4.7) | 1.55 (1.04-2.32) | 1.55 (0.78-3.08) |
| Pacific peoples | 48 (31.0) | 65 (21.4) | 4 (3.9) | 4 (2.1) | 38 (23.3) | 84 (15.0) | | - | - | | 1 (0.7) | 1 (0.2) | 91 (10.7) | 154 (6.8) | 2.25 (1.60-3.16) | 2.34 (1.24-4.41) |
| Others | 2 (1.3) | 6 (2.0) | 16 (15.5) | 18 (9.4) | 3 (1.8) | 6 (1.1) | | 4 (1.4) | 12 (1.6) | | 15 (10.6) | 37 (7.9) | 40 (4.7) | 79 (3.5) | 1.47 (0.97-2.22) | 1.61 (0.80-3.24) |
| **Parity** | | | | | | | | | | | | | | | | |
| Nulliparous | 75 (48.4) | 138 (45.4) | 53 (51.5) | 104 (54.2) | 77 (47.2) | 241 (43.0) | | 165 (57.3) | 296 (40.4) | | 76 (53.5) | 151 (32.3) | 446 (52.4) | 930 (41.2) | 1.77 (1.49-2.11) | 1.86 (1.42-2.44) |
| 1–2 | 56 (36.1) | 138 (45.4) | 35 (34.0) | 83 (43.2) | 66 (40.5) | 283 (50.5) | | 91 (31.6) | 386 (52.7) | | 44 (31.0) | 220 (47.0) | 292 (34.3) | 1110 (49.2) | 1 | 1 |
| 3–4 | 17 (11.0) | 26 (8.6) | 14 (13.6) | 4 (2.1) | 14 (8.6) | 30 (5.4) | | 25 (8.7) | 45 (6.1) | | 17 (12.0) | 71 (15.2) | 87 (10.2) | 176 (7.8) | 1.99 (1.48-2.67) | 1.48 (0.92-2.39) |
| >5 | 7 (4.5) | 2 (0.7) | 1 (1.0) | 1 (0.5) | 6 (3.7) | 6 (1.1) | | 7 (2.4) | 6 (0.8) | | 5 (3.5) | 26 (5.6) | 26 (3.1) | 41 (1.8) | 2.63 (1.57-4.42) | 1.73 (0.76-3.97) |
| **Education** | | | | | | | | | | | | | | | | |
| Primary | 42 (27.1) | 68 (22.4) | 10 (9.7) | 13 (6.8) | 49 (30.1) | 118 (21.1) | | 84 (29.2) | 137 (18.7) | | 2 (1.4) | 12 (2.6) | 187 (22.0) | 348 (15.4) | 1.88 (1.49-2.38) | 1.38 (0.93-2.03) |
| Secondary | 48 (31.0) | 73 (24.0) | 24 (23.3) | 33 (17.2) | 18 (11.0) | 63 (11.3) | | 40 (13.9) | 84 (11.5) | | 31 (21.8) | 90 (19.2) | 161 (18.9) | 343 (15.2) | 1.49 (1.18-1.89) | 1.10 (0.74-1.65) |
| University | 40 (25.8) | 103 (33.9) | 60 (58.3) | 143 (74.5) | 73 (44.8) | 318 (56.8) | | 85 (29.5) | 292 (39.8) | | 70 (49.3) | 213 (45.5) | 328 (38.5) | 1069 (47.4) | 1 | 1 |
| Post-graduate degree | - | - | - | - | 2 (1.2) | 6 (1.1) | | 37 (12.9) | 106 (14.5) | | 34 (23.9) | 128 (27.4) | 73 (8.6) | 240 (10.6) | 1.00 (0.73-1.37) | 1.06 (0.69-1.62) |
| Non-university trade | 25 (16.1) | 60 (19.7) | 2 (1.9) | 1 (0.5) | 20 (12.3) | 52 (9.3) | | 41 (14.2) | 114 (15.6) | | 5 (3.5) | 22 (4.7) | 93 (10.9) | 249 (11.0) | 1.30 (0.98-1.72) | 1.19 (0.78-1.82) |
| **Marital status** | | | | | | | | | | | | | | | | |
| Single | 17 (11.0) | 23 (7.6) | 8 (7.8) | 7 (3.7) | 20 (12.3) | 29 (5.2) | | 40 (13.9) | 71 (9.7) | | 7 (4.9) | 13 (2.8) | 92 (10.8) | 143 (6.3) | 1.90 (1.43-2.52) | 1.59 (1.01-2.52) |
| Married/cohabitating | 138 (89.0) | 281 (92.4) | 91 (88.4) | 183 (95.3) | 143 (87.7) | 531 (94.8) | | 248 (86.1) | 662 (90.3) | | 135 (95.1) | 455 (97.2) | 755 (88.7) | 2112 (93.6) | 1 | 1 |
| **Pre-existing hypertension or diabetes** | | | | | | | | | | | | | | | | |
| No | 152 (98.1) | 300 (98.7) | 101 (98.1) | 191 (99.5) | 158 (96.9) | 549 (98.0) | | 272 (94.4) | 728 (99.3) | | 140 (98.6) | 454 (97.0) | 823 (96.7) | 2222 (98.5) | 1 | 1 |
| Yes | 3 (1.9) | 4 (1.3) | 2 (1.9) | 1 (0.5) | 5 (3.1) | 11 (2.0) | | 16 (5.6) | 5 (0.7) | | 2 (1.4) | 14 (3) | 28 (3.3) | 35 (1.6) | 2.03 (1.21-3.41) | 1.45 (0.65-3.24) |
| **Maternal smoking** | | | | | | | | | | | | | | | | |
| Smoking (beyond first trimester) | 34 (21.9) | 47 (15.5) | 10 (9.7) | 18 (9.4) | 25 (15.3) | 40 (7.1) | | 67 (23.3) | 79 (10.8) | | 9 (6.3) | 21 (4.5) | 145 (17.0) | 205 (9.1) | 2.05 (1.61-2.6) | 1.94 (1.27-2.96) |
| Non-smoker | 121 (78.1) | 257 (84.5) | 93 (90.3) | 174 (90.6) | 138 (84.7) | 520 (92.9) | | 221 (76.7) | 654 (89.2) | | 130 (91.6) | 437 (93.4) | 703 (72.6) | 2042 (90.5) | 1 | 1 |
| **Recreational drug use (during pregnancy)** | | | | | | | | | | | | | | | | |
| No | 142 (91.6) | 292 (96.1) | 100 (97.1) | 188 (97.9) | 160 (98.2) | 549 (98.0) | | 280 (97.2) | 727 (99.2) | | 138 (97.2) | 436 (93.7) | 820 (96.4) | 2192 (97.1) | 1 | 1 |
| Yes | 13 (8.4) | 12 (4.0) | 3 (2.9) | 4 (2.1) | 3 (1.8) | 11 (2.0) | | 8 (2.8) | 6 (0.8) | | 2 (1.4) | 18 (3.9) | 29 (3.4) | 51 (2.3) | 1.49 (0.93-2.38) | 0.60 (0.22-1.61) |
| **Infant birthweight customised centile** | | | | | | | | | | | | | | | | |
| <10^th^ | 56 (36.1) | 27 (9.0) | 21 (20.4) | 15 (7.8) | 42 (25.8) | 67 (12.0) | | 118 (41.1) | 82 (11.2) | | 41 (29.5) | 21 (9.2) | 278 (32.8) | 212 (10.6) | 5.62 (4.12-7.66) | 5.57 (3.52-8.83) |
| 10–24.9^th^ | 21 (13.6) | 43 (14.4) | 20 (19.4) | 29 (15.1) | 26 (16.0) | 77 (13.8) | | 43 (15.0) | 121 (16.6) | | 24 (17.3) | 25 (10.9) | 134 (15.8) | 295 (14.7) | 1.95 (1.41-2.69) | 2.06 (1.27-3.34) |
| 25–49.9^th^ | 31 (20.0) | 77 (25.8) | 18 (17.5) | 49 (25.5) | 34 (20.9) | 136 (24.3) | | 55 (19.2) | 193 (26.4) | | 23 (16.6) | 58 (25.3) | 161 (19.0) | 513 (25.5) | 1.34 (0.99-1.83) | 1.65 (1.04-2.60) |
| 50–74.9^th^ | 20 (12.9) | 80 (26.8) | 14 (13.6) | 61 (31.8) | 35 (21.5) | 132 (23.6) | | 43 (15.0) | 153 (21.0) | | 15 (10.8) | 58 (25.3) | 127 (15.0) | 484 (24.1) | 1.12 (0.82-1.55) | 1.77 (1.11-2.82) |
| 75–89.9^th^ | 13 (8.4) | 45 (15.1) | 14 (13.6) | 21 (10.9) | 11 (6.8) | 100 (17.9) | | 18 (6.3) | 110 (15.1) | | 18 (13.0) | 41 (17.9) | 74 (8.7) | 317 (15.8) | 1 | 1 |
| >90^th^ | 14 (9.0) | 27 (9.0) | 16 (15.5) | 17 (8.9) | 15 (9.2) | 48 (8.6) | | 10 (3.5) | 71 (9.7) | | 18 (13.0) | 26 (11.4) | 73 (8.6) | 189 (9.4) | 1.66 (1.14-2.40) | 1.55 (0.89-2.72) |
| **Fetal movement frequency (last two weeks)** | | | | | | | | | | | | | | | | |
| Increased | 13 (8.4) | 85 (28.0) | 6 (5.8) | 17 (8.9) | 21 (12.9) | 217 (38.8) | | 36 (12.5) | 254 (34.7) | | 7 (4.9) | 75 (16.0) | 83 (9.8) | 648 (28.7) | 0.33 (0.26-0.43) | 0.30 (0.21-0.43) |
| Decreased | 45 (29.0) | 36 (11.8) | 9 (8.7) | 13 (6.8) | 61 (37.4) | 82 (14.6) | | 84 (29.2) | 63 (8.6) | | 66 (46.5) | 74 (15.8) | 265 (31.1) | 268 (11.9) | 2.74 (2.22-3.37) | 2.92 (2.16-3.94) |
| No change/unsure | 97 (62.6) | 183 (60.2) | 88 (85.4) | 162 (84.4) | 81 (49.7) | 261 (46.6) | | 168 (58.3) | 416 (56.8) | | 63 (44.4) | 225 (48.1) | 497 (58.4) | 1247 (55.3) | 1 | 1 |

Data are number (percentage) or median (IQR). TASS=The Auckland Stillbirth Study. SSS=Sydney Stillbirth Study. MCSS=New Zealand Multicentre Stillbirth Study. MiNESS=Midlands and North of England Stillbirth Study. STARS=Study of Trends and Associated Risks for Stillbirth Study. Participants with missing data were excluded from the multivariable models. No imputation for missing data. *South Asian includes India, Pakistan, Sri Lanka, Bangladesh, Nepal, Bhutan, the Maldives, and Afghanistan ethnicities**.** Multivariable models are adjusted for matching terms (gestation at interview or survey in controls and at diagnosis of stillbirth for cases), study and site, age, BMI, ethnicity, parity, education, marital status, pre-existing hypertension or diabetes, smoking, drug use, baby birthweight centile, fetal movement, supine going-to-sleep position, habitual snoring, the Berlin Questionnaire, restless sleep, sleep duration, and daytime naps.

Reprinted from EClinicalMedicine, Vol 10, Authors: Cronin, RS., Li, M., Thompson, JMD., Gordon, A., Raynes-Greenow, CH., Heazell, AEP., Stacey, T., Culling, VM., Bowring, V., Anderson, NH., O'Brien, LM., Mitchell, EA., Askie, LM., McCowan, LME, An Individual Participant Data Meta-analysis of Maternal Going-to-Sleep Position, Interactions with Fetal Vulnerability, and the Risk of Late Stillbirth, Pages 49-57., Copyright (2019), with permission from Elsevier.
